# Different Prognostic Role of Soluble PD-L1 in the Course of Severe and Non-Severe COVID-19

**DOI:** 10.3390/jcm12216812

**Published:** 2023-10-27

**Authors:** Francesco Sabbatino, Pasquale Pagliano, Carmine Sellitto, Berenice Stefanelli, Graziamaria Corbi, Valentina Manzo, Emanuela De Bellis, Luigi Liguori, Francesco Antonio Salzano, Stefano Pepe, Amelia Filippelli, Valeria Conti

**Affiliations:** 1 Department of Medicine, Surgery and Dentistry “Scuola Medica Salernitana”, University of Salerno, 84081 Baronissi, Italy; fsabbatino@unisa.it (F.S.); ppagliano@unisa.it (P.P.); csellitto@unisa.it (C.S.); bstefanelli@unisa.it (B.S.); vmanzo@unisa.it (V.M.); edebellis@unisa.it (E.D.B.); frsalzano@unisa.it (F.A.S.); spepe@unisa.it (S.P.); afilippelli@unisa.it (A.F.); vconti@unisa.it (V.C.); 2 Oncology Unit, San Giovanni di Dio e Ruggi D’Aragona University Hospital, 84131 Salerno, Italy; 3 Infectious Disease Unit, San Giovanni di Dio e Ruggi D’Aragona University Hospital, 84131 Salerno, Italy; 4 Clinical Pharmacology Unit, San Giovanni di Dio e Ruggi d’Aragona University Hospital, Via San Leonardo 1, 84131 Salerno, Italy; 5 Department of Translational Medical Sciences, University of Naples “Federico II”, 80131 Naples, Italy; 6 Department of Clinical Medicine and Surgery, University of Naples “Federico II”, 80131 Naples, Italy; luigiliguori1992@gmail.com; 7 Otolaryngology Unit, San Giovanni di Dio e Ruggi D’Aragona University Hospital, 84131 Salerno, Italy

**Keywords:** COVID-19, PD/PD-L1 axis, sPD-L1, cytokine storm, ARDS, length of stay, immune response, inflammation

## Abstract

Understanding the link between COVID-19 and patient immune characteristics is crucial. We previously demonstrated that high levels of the soluble Programmed Death-Ligand1 (sPD-L1) at the beginning of the infection correlated with low lymphocyte number and high C-reactive protein (CRP), longer length of stay (LOS), and death. This study investigated whether sPD-L1 can be a prognosis biomarker during COVID-19. Severe and non-severe COVID-19 patients were enrolled at the University Hospital of Salerno. During hospitalization, at admission, and after 12–14 days, patients’ data were collected, and sPD-L1 levels were measured by enzyme-linked immunosorbent assay. The peripheral lymphocyte number negatively correlated with the time of negativization (*p* = 0.006), length of stay (LOS) (*p* = 0.032), and CRP (*p* = 0.004), while sPD-L1 positively correlated with LOS (*p* = 0.015). Patients with increased sPD-L1 and lymphocyte number showed a shorter LOS than those with decreased sPD-L1 and lymphocyte number (*p* = 0.038) and those with increased sPD-L1 and decreased lymphocyte number (*p* = 0.025). Moreover, patients with increased sPD-L1 and decreased CRP had a shorter LOS than those with increased sPD-L1 and CRP (*p* = 0.034) and those with decreased sPD-L1 and CRP (*p* = 0.048). In conclusion, while at an early phase of COVID-19, sPD-L1 promotes an immune escape, later, it might act to dampen an excessive immune response, proving its role in COVID-19 prognosis.

## 1. Introduction

Despite significant reductions in the number of clinical cases and advances in treatment, COVID-19 syndrome continues to cause hospitalizations and deaths, posing a threat to human health worldwide [1]. Especially at the beginning of the pandemic, in the absence of specific medicines and guidelines, repurposed drugs were often used, even when scientific evidence was limited [2,3]. Although anti-COVID-19 molecules are finally available, numerous clinical trials and basic research are ongoing to find prognostic markers to optimize treatment. In this context, it is crucial to understand the link between patients’ immune characteristics and the severe manifestations of COVID-19, characterized by dysregulation of both innate and adaptive immune responses [4].

Significant hematologic changes occur in COVID-19 patients involving well-known biomarkers, such as number of lymphocytes, neutrophils and platelets, and platelet/lymphocyte ratio. In addition, significant changes occur in the levels of soluble mediators of inflammation, including interleukin (IL)-2 and -6, interferon-induced protein 10 (IP-10), monocyte chemoattractant protein (MCP-3), and macrophage colony-stimulating factor (M-CSF). Consequently, the combination of hematologic and inflammatory parameters with levels of lactate dehydrogenase (LDH) and C-reactive protein (CRP) has proven useful in determining disease severity [5].

COVID-19 is characterized by the so-called “cytokine storm”, abnormal activation of different cell types and persistent production of pro-inflammatory cytokines, which alters differentiation, proliferation, and activation of various immune cells, as well as the polarization of CD4+ and CD8+ T lymphocytes, resulting in serious and life-threatening complications [6,7].

Most of the COVID-19 patients are elderly. Aging is known to be related to chronic low-grade inflammation (i.e., inflammaging) [8]. Therefore, it is not surprising that several age-associated factors involved in the homeostatic response to inflammatory stimuli have been recognized to influence the severity of COVID-19 [9,10,11].

The immune checkpoints programmed death-1 (PD-1) and its ligand programmed death-ligand 1 (PD-L1) play a crucial role in maintaining the balance between stimulation and activation of immune cells [12]. They work through enhancing T-cell tolerance and T-cell depletion and increasing the immunosuppressive function of regulatory T-cells (TREG) [13,14]. The study of the PD-1/PD-L1 axis in tumors, which are effectively able to evade the guard of the immune system, has led to the possibility of improving cancer treatment using immunotherapeutic agents such as anti-PD-1/PD-L1 monoclonal antibodies, commonly called immune checkpoint inhibitors (ICIs) [15]. There are different forms of PD-L1: transmembrane (mPD-L1), exosomal (exoPD-L1), nuclear (nPD-L1), and soluble (sPD-L1) [16]. Several studies have reported high expression levels of mPD-L1 in cancer cells, so it has been proposed as a predictive biomarker of response to immunotherapy [16,17]. Unlike their cellular counterparts, the role of soluble forms of PD1 and PD-L1 (sPD-1 and sPD-L1) has not yet been well elucidated. Accumulating evidence has shown that high plasma levels of sPD-1 and sPD-L1 may be related to poor prognosis and worse therapeutic outcomes in cancer patients, representing possible biomarkers of tumor pathogenesis and prognosis [18,19]. Notably, high and persistent expression of sPD-L1 has been reported not only in tumors but also in autoimmune and viral diseases [16]. We previously proposed sPD-L1 as a valuable prognosis biomarker for COVID-19, having found that high levels of sPD-L1 correlated with low lymphocyte counts and high CRP levels and were also associated with longer hospital length of stay (LOS) and death. Furthermore, the upregulation of sPD-L1 was induced by SARS-CoV-2 in infected epithelial cells [20]. In another observational study, sPD-L1 upregulation was reported in COVID-19 patients who required invasive mechanical ventilation [21]. Given the dynamic nature of sPD-L1 and its role as an immune modulator, altered levels of sPD-L1 likely reflect changes in the host’s immune and inflammatory response during infection. Therefore, the present study aimed to monitor how sPD-L1 levels change in COVID-19 patients during hospitalization and to explore whether these changes could correlate with clinical outcomes and biochemical and clinical parameters.

## 2. Materials and Methods

### 2.1. Study Population

Patients diagnosed with COVID-19 were consecutively enrolled at the infectious disease Unit of the University Hospital of Salerno from October 2021 to March 2022.

All recruited subjects presenting for COVID-19 suspicion were Caucasians aged 18 years or older and had undergone a nasopharyngeal swab for SARS-CoV-2-RNA at the time of hospital admission to confirm the diagnosis. Moreover, all were presented with characteristic infiltrates observed by a chest CT scan (bilateral presence of patchy ground glass opacities that may coalesce into dense, consolidative lesions, with a predominantly peripheral distribution under the pleura and along the broncho-vascular bundles).

Nasopharyngeal swabs were performed, stored, and delivered to the testing laboratory as recommended by the CDC and ECDC. Nasopharyngeal swabs were performed following a standardized procedure. Briefly, GeneFinder™ COVID-19 PLUS RealAmp Kit has been used for the detection of SARS-CoV-2 virus through reverse Transcription and Real-Time Polymerase Chain Reaction from RNA extracted from nasopharyngeal swab (ELITe InGenius^®^system; ELITechGroup, Puteaux, France). The extraction volume was 200 μL. One-Step Reverse Transcription Real-Time polymerase chain reaction is used to confirm the presence of COVID-19 by amplification of RdRp, E, and N genes. The cut-off Ct value of the GeneFinder COVID-19 Plus RealAmp Kit (ELITechGroup, Puteaux, France) assay is 40, and the analytical sensitivity of the assay is 1 copy/μL.

All participants signed informed consent. The study was approved by the local Ethics Committee (n.30_r.p.s.o./2020) in accordance with the Declaration of Helsinki and its amendments and was carried out without interfering with normal clinical practice.

Patient data, including comorbidities, need for high-flow oxygen therapy, in-hospital LOS, and time to SARS-CoV-2 negativization, as well as data on treatment, death, or hospital discharge, were retrieved from medical records.

The number of peripheral blood cells (neutrophils, lymphocytes, and platelets), C-reactive protein (CRP), lactate dehydrogenase (LDH), erythrocyte sedimentation rate (ESR), D-Dimer, ferritin, fibrinogen, and arterial oxygen partial pressure/fractional inspired oxygen (PaO_2_/FiO_2_), and other biochemical and functional parameters were routinely collected during hospital length of stay.

### 2.2. Collection of Blood Samples and Determination of the Amount of Soluble PD-L1

Venous blood samples were collected from each patient at the same time at which biochemical and functional parameters were collected: at the time of admission (T0) and after 12–14 days from admission (T1).

These samples were centrifuged for 15 min at 1000× *g* at 2–8 °C to obtain plasma that was immediately stored at −80 °C until analysis. Levels of sPD-L1 were determined by enzyme-linked immunosorbent assay (ELISA) following the manufacturer’s instructions (Elabscience Biotechnology Co. Ltd., Wuhan, China).

Specifically, the following steps were followed: addition of standards or samples (100 μL) to the wells of the micro ELISA plate pre-coated with an antibody specific for human PD-L1 and incubation for 90 min at 37 °C; addition of biotinylated detection antibody working solution (100 μL) to each well and incubation for 60 min at 37 °C; wash 3 times; addition of horseradish peroxidase (HRP) conjugate (100 μL) working solution (100 μL) with incubation for 30 min at 37 °C; wash 5 times; addition of 90 μL substrate reagent and incubation for 15 min at 37 °C. Finally, the enzyme-substrate reaction was terminated by adding the stop solution (50 μL).

Optical density (OD value) was measured by spectrophotometry analysis using a microplate reader (TECAN^®^ infinite 200 PRO, Männedorf, Switzerland) set at 450 nm. Each sample was analyzed in duplicate, and the level of sPD-L1 was measured using a four-parameter logistic curve. The sensitivity of the assay was 0.10 ng/mL with a detection range of 0.16–10 ng/mL. The intra-assay and inter-assay coefficients of variation were less than 10%. Results are expressed as mean ± SD of three independent experiments.

### 2.3. Statistical Analysis

Statistical analysis was performed using the Stata Statistical Software, Version 16.0 (StataCorp. Stata Statistical Software: Release 16. College Station, TX: StataCorp LLC, College Station, TX, USA). Correlations between pathological and clinical characteristics with levels of biohumoral parameters of COVID-19 patients, including sPD-L1, were analyzed by the Spearman rank correlation test. Differences in the expression levels of variables according to pathological and clinical characteristics were analyzed using the Mann–Whitney U test. The correlation of the differences in levels of biohumoral parameters at different times (∆ = T_0_ − T_1_) with clinical outcomes such as the number of deaths, time to SARS-CoV-2 negativization, and LOS were analyzed by Fisher exact test or Mann–Whitney U test, as appropriated. Multiple logistic and linear regression analyses were performed to evaluate the association among variables considering the possible confounders.

All the analyses were first performed as univariate evaluations and then adjusted for age and sex/gender. All Figures show the adjusted results.

A *p*-value below 0.05 was considered statistically significant. All tests used were two-tailed.

## 3. Results

### 3.1. Patient Characteristics

The study population consisted of 30 COVID-19 patients (33.0% with severe and 67.0% with non-severe disease). Clinical characteristics and laboratory data collected at hospital admission (T_0_) and after 12–14 days (T_1_) are reported in Table 1. COVID-19 severity was established based on the PaO_2_/FIO_2_ ratio < 300 and symptomatology of patients at the hospital admission.

The most prevalent comorbidities were hypertension, followed by cardiovascular disease, diabetes, neoplasms, chronic kidney disease, obesity, dyslipidemia, neurologic diseases, liver and biliary tract diseases, and chronic pulmonary disease. At baseline, no differences in medical therapy were found, and no statistically significant changes occurred during the study period. Drugs used for COVID-19 treatment included corticosteroids, and low molecular weight heparin (LMWH) was the most used, followed by azithromycin, tocilizumab, and casivirimab/indevimab. The mean LOS was 34.6 days (range, 12–74), while the mean time to SARS-CoV-2 negativization was 34.8 days (range, 13–73). Four patients (13.3%) died from Acute Respiratory Distress Syndrome (ARDS), while 26 patients (86.7%) were discharged home.

### 3.2. Correlation between Baseline Biohumoral Characteristics of COVID-19 Patients with Their Clinical Outcomes

By the univariate analysis, the number of peripheral lymphocytes was significantly higher in discharged than in deceased patients (*p* = 0.05). CRP and LDH levels were higher in deceased patients than in discharged patients (*p* = 0.0268 and *p* = 0.0235, respectively). In addition, the PaO_2_/FiO_2_ ratio was significantly lower in deceased patients as compared to discharged patients (*p* = 0.0050).

**Figure 1:**
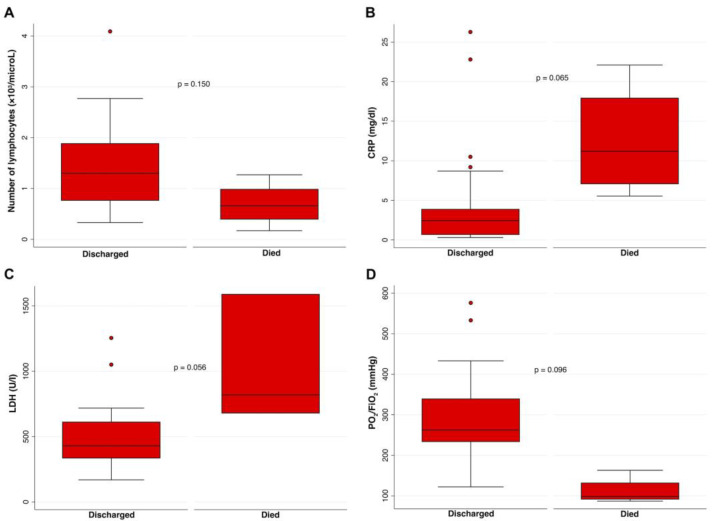
Association between (**A**) the number of peripheral lymphocytes, (**B**) the CRP levels, (**C**) the LDH levels, and (**D**) the PaO_2_/FiO_2_ levels in COVID-19 patients who died or were discharged from the hospital were compared using a multivariate logistic regression, adjusting for age and sex/gender. On each box, the central mark is the median, the edges of the box are the 25th and 75th percentiles, the whiskers extend to the most extreme data points not considered outliers, and outliers are plotted individually. *p* was considered significant if <0.05. CRP, C-Reactive Protein; LDH, lactate dehydrogenase; PaO_2_/FiO_2_, arterial oxygen partial pressure/fractional inspired oxygen.

After adjustment for age and sex/gender, although without reaching a statistical significance, the number of peripheral lymphocytes was higher in discharged than in deceased patients (Figure 1A). CRP and LDH levels were higher in deceased patients than in discharged patients (Figure 1B,C). In addition, the PaO_2_/FIO_2_ ratio was lower in deceased patients as compared to discharged patients (Figure 1D). All the associations were adjusted for age and sex/gender.

In the univariate analysis, there was a direct correlation between LOS and time to SARS-CoV-2 negativization (*p* < 0.0001). The number of peripheral lymphocytes significantly correlated with both time to SARS-CoV-2 negativization (*p* = 0.0029) and LOS (*p* = 0.0256). Lastly, sPD-L1 levels positively correlated with LOS (*p* = 0.0078). Specifically, patients displaying a higher level of sPD-L1 at the time of hospital admission had a longer LOS.

**Figure 2:**
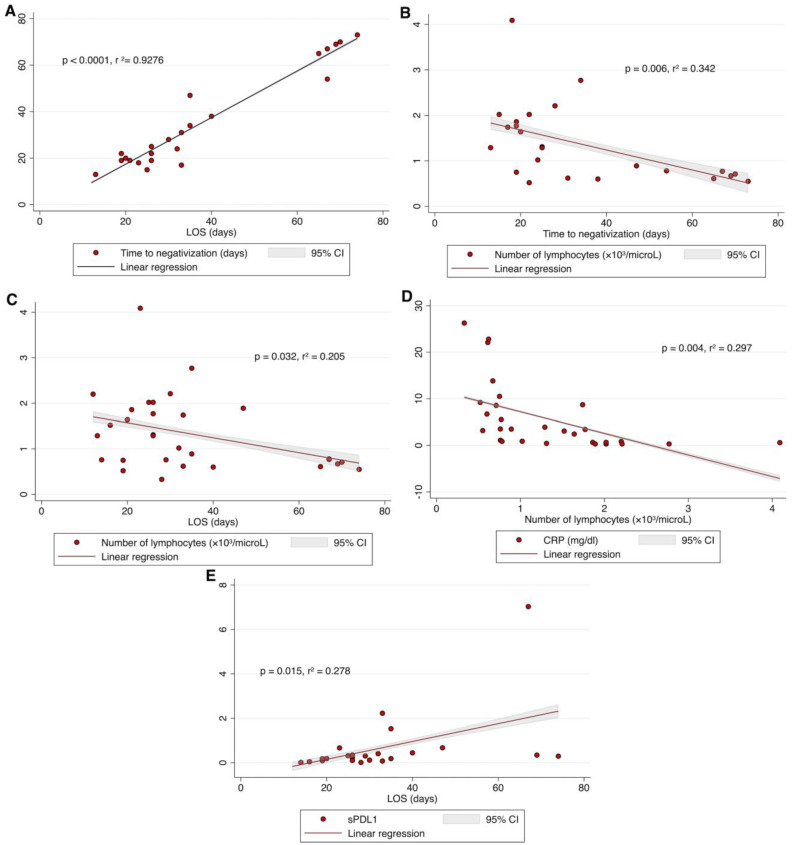
The time to SARS-CoV-2 negativization was positively correlated with LOS (**A**); the number of peripheral lymphocytes was negatively correlated with the time to negativization (**B**), and with LOS (**C**); CRP levels were negatively correlated with number of peripheral lymphocytes (**D**); sPD-L1 levels positively correlated with LOS (**E**). All data were analyzed by linear regression, adjusted for age and sex/gender; *p* was considered significant if <0.05. LOS, length of hospital stay; CRP, C-Reactive Protein; sPD-L1, soluble PD-L1.

After adjustment for age and sex/gender, as shown in Figure 2A, there was a direct correlation between LOS and time to SARS-CoV-2 negativization (*p* < 0.0001, *r*^2^ = 0.928). The number of peripheral lymphocytes significantly correlated with both time to SARS-CoV-2 negativization (*p* = 0.006, *r*^2^ = 0.342, Figure 2B) and LOS (*p* = 0.032, *r*^2^ = 0.205, Figure 2C). Specifically, patients displaying a lower number of lymphocytes had a longer time to SARS-CoV-2 negativization (Figure 2B) as well as a longer LOS (Figure 2C). Moreover, as shown in Figure 2D, the number of peripheral lymphocytes negatively correlated with CRP levels (*p* = 0.004, *r*^2^ = 0.297). Lastly, sPD-L1 levels positively correlated with LOS (*p* = 0.015, *r*^2^ = 0.278). Specifically, patients displaying a higher level of sPD-L1 at the time of hospital admission had a longer LOS (Figure 2E).

### 3.3. Correlation between Differences in Biohumoral Characteristics during Hospitalization of COVID-19 Patients with Clinical Outcomes

By considering the differences in levels of biohumoral parameters at different times (∆ = T0 − T1), the number of peripheral lymphocytes, as well as sPD-L1 levels, still significantly correlated with LOS. There was a significant difference in LOS between patients whose lymphocytes or sPD-L1 increased or decreased during time: patients in whom the number of lymphocytes increased during hospitalization displayed a lower LOS as compared to that of patients with a decrease (*p* = 0.0341). In patients in whom sPD-L1 levels increased during hospitalization, a shorter LOS (*p* = 0.0136) was found as compared to that with a decrease in sPD-L1 levels increased.

**Figure 3:**
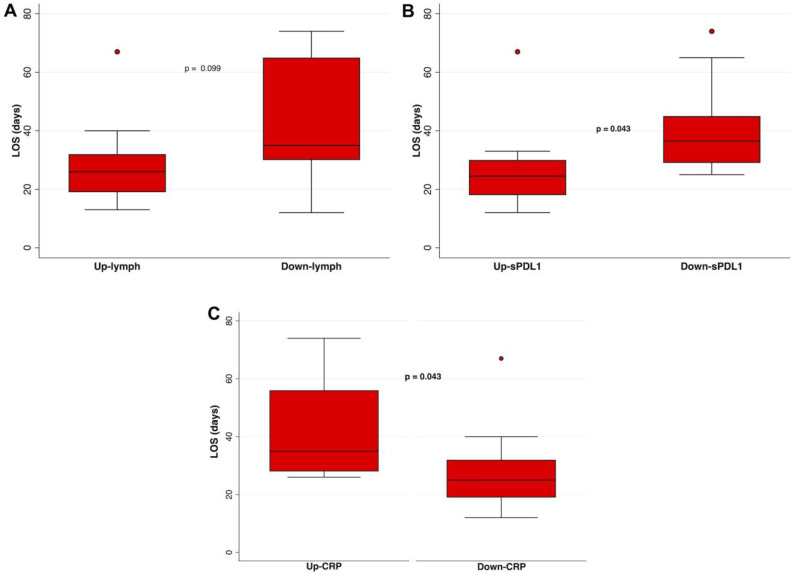
Association between the LOS in patients in whom (**A**) the number of lymphocytes increased (Up-lymphs) or decreased (Down-lymphs), (**B**) the sPD-L1 levels increased (Up-sPD-L1) or decreased (Down-sPD-L1), (**C**) the CRP levels increased (Up-CRP) or decreased (Down-CRP) during hospitalization were compared using multivariate logistic regression analysis, adjusted for age and sex/gender. On each box, the central mark is the median, the edges of the box are the 25th and 75th percentiles, the whiskers extend to the most extreme data points not considered outliers, and outliers are plotted individually. *p* was considered significant if <0.05. sPD-L1, soluble PD-L1; LOS, length of hospital stay; lymphs, lymphocytes.

After adjustment for age and sex/gender, we found that the number of peripheral lymphocytes was not correlated with LOS (Figure 3A). The CRP levels and sPD-L1 levels significantly correlated with LOS. There was a significant difference in LOS between patients whose CRP or sPD-L1 levels increased or decreased over time. Specifically, in patients in whom sPD-L1 levels (Up-sPD-L1) increased during hospitalization, a shorter LOS was found as compared to those with a decrease in sPD-L1 levels (Figure 3B, *p* = 0.043). Lastly, in patients in whom CRP levels (Up-CRP) decreased during hospitalization, a shorter LOS was found as compared to those with an increase in CRP levels (Figure 3C, *p* = 0.043). All the correlations were adjusted for age and sex/gender.

In the univariate analysis, by stratifying patients based on the combination of increasing or decreasing sPD-L1 levels, number of lymphocytes, and CRP levels, four prognostic groups of COVID-19 patients were identified based on their LOS. Notably, patients belonging to the group with increasing sPD-L1 and number of lymphocytes, as well as those belonging to the group with increasing sPD-L1 but decreasing CRP levels, had a LOS significantly shorter than the other groups (*p* = 0.0139 and *p* = 0.0297, respectively).

**Figure 4:**
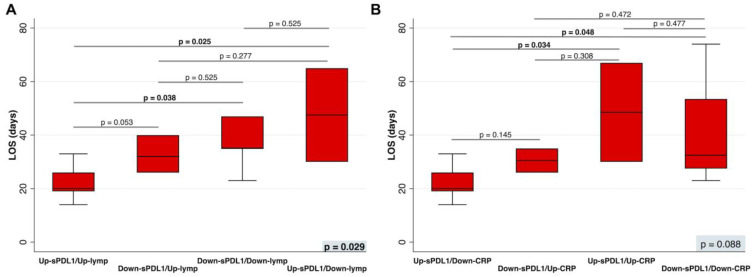
(**A**) The LOS in patients in whom the sPD-L1 levels increased (Up-sPD-L1) or decreased (Down-sPD-L1) and the number of lymphocytes increased (Up-lymphs) or decreased (Down-lymphs), or (**B**) the CRP levels increased (Up-CRP) or decreased (Down-CRP) during hospitalization were compared by a multinominal logistic regression, adjusted for age and sex/gender. On each box, the central mark is the median, the edges of the box are the 25th and 75th percentiles, the whiskers extend to the most extreme data points not considered outliers, and outliers are plotted individually. *p* was considered significant if <0.05. LOS, length of hospital stay; sPD-L1, soluble PD-L1; lymphs, lymphocytes; CRP, C-Reactive Protein.

After adjustment for age and sex/gender, patients belonging to the group with increasing sPD-L1 and number of lymphocytes (Figure 4A), as well as those belonging to the group with increasing sPD-L1 but decreasing CRP levels (Figure 4B), had a LOS significantly shorter than Down-sPDL1/Down-Lymph (*p* = 0.038), and Up-sPDL1/Up-CRP (*p* = 0.034). Moreover, Up-sPDL1/Up-Lymph had a LOS significantly shorter than Up-sPDL1/Down-Lymph (*p* = 0.025). Similarly, Up-sPDL1/Up-CRP had a LOS significantly shorter than Up-sPDL1/Down-CRP (*p* = 0.048). All analyses were adjusted for age and sex/gender.

## 4. Discussion

Understanding the link between patients’ immune characteristics and the severity of COVID-19 is a critical step not only to counter this syndrome but also to improve our knowledge of the functioning of the immune system and set up more effective therapy against many viral infections [22]. We have previously shown that sPD-L1 could be a valuable biomarker of prognosis in COVID-19 patients [20]. Patients with high mean sPD-L1 content were characterized by low lymphocyte counts and elevated CRP levels. In addition, higher levels of sPD-L1 were found in patients with a longer LOS and in all patients who died compared with those who were discharged [20].

Given the dynamic nature of sPD-L1, in the present study, we examined whether changes in sPD-L1 could correlate with clinical outcomes, including LOS and death, as well as with biochemical and clinical parameters of COVID-19 patients.

Baseline sPD-L1 levels correlated positively with LOS (*p* = 0.015, *r*^2^ = 0.278, Figure 2E), confirming the main finding of the previous study. In contrast, by monitoring sPD-L1 in this study, we found that patients in whom sPD-L1 increased during hospitalization (Up-sPD-L1) had a shorter LOS (*p* = 0.043, Figure 3B) than those who showed a downward trend in sPD-L1 (Down-sPDL1).

Beyond cancer, the role of mPD-L1 and sPD-L1 and the use of ICIs have been studied in patients with chronic infections such as HIV, HBV, and HCV [23,24,25,26,27,28]. In addition, the involvement of the PD-1/PD-L1 axis has been suggested in the pathogenesis of long COVID-19, in which a depletion of CD4+ and CD8+ T-cells was found [29]. The recognized involvement of the PD-1/PDL1 axis in cancer and during infections has provided the incentive to investigate the role of these immune response controllers in COVID-19, however, leading to conflicting and inconclusive results [30,31,32,33,34]. Moritz et al., in a study on 13 COVID-19 patients affected by melanoma on Immune Checkpoint Inhibition (ICI) treatment, reported that only 2 patients were hospitalized [30]. Accordingly, Luo et al. found no significant association between ICI treatment and severity of COVID-19 in patients with lung cancer [32]. Qian et al. meta-analyzed 13 studies, including a total of 4134 cancer patients with COVID-19, finding an increased rate of hospitalization in patients who used ICIs compared with those who did not [35].

By combining sPDL-1 levels with the number of peripheral lymphocytes and sPDL-1 levels with CRP levels, we highlighted the dynamic role of sPDL-1 in modulating the immune and inflammatory response during COVID-19. High sPD-L1 levels, initially associated with poor patient prognosis, later predicted better outcomes. In fact, compared with baseline, a shorter LOS was observed in patients in whom both sPD-L1 levels and lymphocyte count increased, demonstrating recovery of lymphopenia, and in those in whom the increase in sPD-L1 was accompanied by an attenuation of the inflammatory state demonstrated by decreased CRP levels (Figure 4A,B).

We suggest that the different correlations found at baseline and during hospitalization between sPD-L1 levels and clinical outcomes likely reflect the different levels of sPD-L1 produced during infection.

Indeed, we previously reported [20] that SARS-CoV-2 can induce overexpression of mPD-L1 from infected cells as an immune escape mechanism. Accordingly, during an early phase of COVID-19 disease, these cellular levels reflect those of the soluble form, sPD-L1. In contrast, we have also shown that mPD-L1 levels are dysregulated on different types of immune cells in patients with ARDS [20]. Therefore, in the second phase of the disease, sPD-L1 levels could reflect a mechanism used to regulate and dampen an excessive host immune response, which could lead to the so-called cytokine storm and severe tissue damage. However, further experiments are needed to elucidate the mechanisms underlying sPD-L1 production and to define the different prognostic roles of sPD-L1 during different stages of COVID-19 syndrome.

Several in vivo studies corroborate the finding, suggesting that PD-L1 is required to regulate both innate and adaptive immune responses to counteract excessive inflammation. In nonobese diabetic (NOD) mice, knockout of PD-L1 or PD-1 has been observed to determine the development of autoimmune diabetes [36,37]. Moreover, PD-L1 has been shown to induce the development and activity of TREG, blocking excessive activation of the immune system in response to viral infections [38,39]. A recent study has demonstrated that administration of sPD-L1 in mice with ARDS alleviated inflammatory lung damage and improved survival rate by decreasing the number of lung monocyte-derived macrophages and their pro-inflammatory markers [40]. Monaghan et al. showed that splenocytes cultured with bronchial alveolar lavage (BAL) from mice with ARDS and containing PD1 and its ligand have lower production of TNF-α than those cultured with BAL lacking sPD-1, suggesting that binding of sPD-1 to PD-1 may have an anti-inflammatory effect [41]. The changes in sPD-L1 levels detected in COVID-19 over time explain the complexity of the immune response developed and perpetuated during COVID-19 and underscore the importance of balanced regulation of immunoregulatory factors to avoid an over-response strongly associated with cytokine storm.

This study has some limitations. First, the small number of deaths (n.4) observed did not allow us to find a correlation between sPD-L1 and death. In addition, we assessed the inflammatory status of patients by monitoring only CRP values without measuring levels of cytokines with a recognized role in COVID-19, such as IL-6.

## 5. Conclusions

Although we would have gladly done without it, the COVID-19 pandemic has represented a learning opportunity.

Our results confirm the involvement of sPD-L1, such an important molecule in modulating both innate and adaptive immune response and demonstrate its role in the prognosis of COVID-19.

This study represents the first evidence of the role of the soluble form of PDL1 as a possible parameter to follow the progression of COVID-19 disease, but also to check the therapy effectiveness. Further research should be addressed to better define the differences between the distinctive PDL1 forms in the pathogenesis, progression, and prognosis not only during COVID-19 but also for other immune diseases.

Improving our knowledge of the mechanisms underlying immune system function could be useful in the future to counter not only COVID-19 but also other conditions associated with dysregulation of the host immune response and to monitor patient outcomes in response to treatment.

## Figures and Tables

**Table 1:** Patients’ characteristics and medication use at baseline (T_0_) and after 12–14 days (T_1_).

Characteristics of the Study Population	T_0_	T_1_
Age, years (range)	66.3 (42–84)	
Sex, *n* (%):		
Male	20 (66.7)	
Female	10 (33.3)	
Mean number of PBMCs:		
White blood cells count, ×10^3^/µL (range)	7.9 (2.47–19.04)	8.0 (2.77–15.42)
Lymphocytes, ×10^3^/µL (range)	1.3 (0.17–4.09)	1.5 (0.4–2.72)
Platelets, ×10^3^/mL (range)	226.9 (74–568)	235.3 (26–414)
LDH, U/I (range)	557.9 (169–1592)	604.3 (204–2957)
ESR, mm (range)	35.7 (16–75)	33.4 (2–50)
CRP, mg/dL (range)	5.67 (0.3–26.27)	2.5 (0.15–14.77)
Fibrinogen, mg% (range)	535.7 (210–908)	518.6 (296–1016)
PaO_2_/FiO_2_ ratio	258.9	272.5
Severe	10 (33.0)	
Non-severe	20 (67.0)	
Chronic diseases, *n* (%):		
Hypertension	14 (46.7)	
Cardiovascular diseases	14 (45.0)	
Diabetes	8 (26.7)	
Previous neoplasms	5 (16.7)	
Obesity	4 (13.3)	
Chronic kidney disease	4 (13.0)	
Dyslipidemia	3 (10.0)	
Neurologic diseases	2 (6.7)	
Liver and biliary tract diseases	2 (6.7)	
Chronic pulmonary disease	1 (3.0)	
Medium LOS, days (range)	34.6 (12–74)	
Mean time to SARS-CoV-2 negativization, days (range)	34.8 (13–73)	
Therapy, *n* (%):		
Low molecular weight heparin	12 (40.0)	
Corticosteroids	11 (36.6)	
Azithromycin	4 (13.3)	
Tocilizumab	2 (6.6)	
Casivirimab/Indevimab	1 (3.3)	
Number of deaths (%)	4 (13.3)	

Abbreviations: PBMCs, Peripheral Blood Mononuclear Cells; LDH, lactate dehydrogenase; ESR, erythrocyte sedimentation rate; CRP, C-reactive protein; LOS, length of hospital stay; PaO_2_/FiO_2_, arterial oxygen partial pressure/fractional inspired oxygen.

## Data Availability

Not applicable.
